# Conceptualizing an antiracist framework for neuroscience research in art therapy: a qualitative pilot study

**DOI:** 10.3389/fnhum.2025.1492779

**Published:** 2025-04-23

**Authors:** Kerry A. Kruk-Borisov

**Affiliations:** Expressive Therapies, Lesley University, Cambridge, MA, United States

**Keywords:** antiracism, art therapy, antiracist neuroscience research, diversity, positionality, antiracist research practices, critical qualitative research

## Abstract

**Introduction:**

Advances in social cognitive neuroscience research have contributed deeper understanding of neural processes relevant to art therapy, and of social, interrelational phenomena including racism and implicit bias. Confoundingly, emerging critical discourse about neuroscience research design highlighted systemic racism, implicit bias, and inequality perpetuated by imaging technologies, lack of diversity, and funding disparities. Emphasis toward antiracist practices within cognitive neuroscience research and various other fields has grown; however, literature on antiracist research practices within art therapy research is scant.

**Methods:**

The purpose of this qualitative pilot research study was to elicit conceptualizations about antiracist research practices from art therapy researchers in response to relevant literature. Purposive sampling was used to recruit four female art therapy researchers from the United States (U.S.) and Europe. Semi-structured interviews were analyzed using grounded theory coding resulting in three main categories, seven themes, and subthemes. Member-checking and reflexive journaling were employed to enhance credibility.

**Results:**

Core categories revealed points of convergence across participants, areas of concern, and requirements outlining antiracist research practices in art therapy. The first core category, *shared beliefs and values*, had three themes: *neuroscience-informed perspective of art therapy; neuroscience research can strengthen art therapy theories*; and *infusion of antiracism and neuroscience into art therapy begins with education*. The second core category, *barriers and challenges*, had two themes: *potential credibility and legitimacy concerns for art therapy;* and *difficult conversations about disparities in awareness, diversity, and resources*. The third core category, *requirements and responsibilities for antiracist research*, had two themes: *due diligence to build accountability and legitimacy*; and *inclusion of diversity in art therapy research*, and subthemes.

**Discussion:**

Preliminary outcomes revealed ideas aligning current antiracist neuroscience research discourse with art therapy experimental research practices. The small group of neuroscience-focused art therapist researchers provided realistic considerations about amplifying discourse within the art therapy profession and infusing antiracist research into neuro-informed art therapy curriculum, and prioritizing diversity throughout experimental research design. An antiracist art therapy research framework with principles including education, intentionality, and diversity was proposed, along with recommendations for further research using the framework and to implement the framework into graduate art therapy education.

## 1 Introduction

Art and creative processes, such as music making and art making, have been explored as unique human phenomena that are transformative, restorative, curative, and rehabilitative activities (deWitte et al., 2019; Karkou et al., 2022; Nummenmaa and Hari, 2023; Oliva et al., 2023; Vaisvaser, 2021), and are connected to cultural identities and that transcend cultural differences (Griffith and Semlow, 2020). Neuroscientific research into creativity and the therapeutic impact of the arts has resulted in new disciplines, such as neuroaesthetics (Chatterjee and Vartanian, 2014; Zeki et al., 2020), Neuroarts which expands on the neuroscience of art (Siler, 2015), and arts in health initiatives (Fancourt and Finn, 2019; Van Lith and Spooner, 2018). Advancements in social cognitive neuroscience research have enhanced in-depth research into neural correlations for emotion and emotional regulation (Braunstein et al., 2017; Gu et al., 2018; Viviani, 2013), empathy (Stevens and Taber, 2021; Zaki and Ochsner, 2012), trauma (Ressler et al., 2022), and the impact on brain functionality and activity as a result of psychological therapies (Abbass et al., 2014; Cozolino, 2024). The application of such research has direct implications for clarifying and refining therapeutic mechanisms and therapeutic approaches across mental health disciplines to include the creative arts therapies (Cozolino, 2024; deWitte et al., 2019; Gu et al., 2018).

### 1.1 Neuroscience research applications in art therapy research

Across the field of creative arts therapies (CAT), and specifically art therapy, neuroscience and neurobiological research have been vital to support fundamental theories and elucidate potential mechanisms of transformation within therapeutic art making processes (King et al., 2019; Lusebrink, 2004, 2014; Malik, 2022; Vaisvaser, 2021). Art therapy employs experiential and somatosensory interventions to stimulate cognitive, emotional, perceptual, and integrative processes for therapeutic and transformative effects (Kapitan, 2012; Vaisvaser, 2021) consistent with theoretical orientations (e.g., psychodynamic, humanistic, narrative, gestalt, etc.) in the broader psychotherapy context (Van Lith, 2016). Researchers have consistently sought to extrapolate more clearly the therapeutic mechanisms of artmaking that lead to change (deWitte et al., 2019; Gerber et al., 2018; Gerge et al., 2019) and to demonstrate the effectiveness of art therapy treatment (Regev and Cohen-Yatziv, 2018; Reynolds et al., 2000; Slayton et al., 2010).

#### 1.1.1 Theoretical applications of neuroscience in art therapy

The application of neuroscience principles and knowledge of brain and body processes has informed the development of fundamental theories of treatment approaches in art therapy practice (Lusebrink, 2004, 2014; Lusebrink, 2010; Hinz, 2019; Czamanski-Cohen and Weihs, 2016; Hass-Cohen and Clyde Findlay, 2015; Vaisvaser, 2021). Researchers have proposed theoretical perspectives which postulate transformative elements within art therapy processes based on neurobiological processes (e.g., stress reduction, perception and insight change, emotional regulation, etc.) to include the Expressive Therapies Continuum (ETC; Hinz, 2019), the bodymind model (Czamanski-Cohen and Weihs, 2016), art therapy relational neuroscience (ATR-N; Hass-Cohen and Clyde Findlay, 2015), and adaptive response theory (ART; Kaimal, 2019). These theoretical perspectives of the underlying mechanics of creative processes incorporate cognitive neuroscience research findings from relevant studies about brain mapping and neural correlations, emotion, cognition, somatosensory functions, creativity, arousal, stress responses, and human information processing which are postulated to be involved in, and intrinsic for, art making for therapeutic impact on a variety of physical and mental disorders (Dietrich and Kanso, 2010; Gu et al., 2018; Oliva et al., 2023; Zaidel, 2010).

#### 1.1.2 Experimental applications of neuroscience in art therapy

For the last two decades, an emerging subset of experimental research across scientific art therapy research studies infused neuroimaging and physiological measurements into scientific exploration to more closely examine internal processes and the impact during art making processes to build scientific evidence (Kaiser and Deaver, 2013; Kapitan, 2012, 2014; King et al., 2019; King and Kaimal, 2019; Malik, 2022; Vaisvaser, 2021), and to fortify those theories that underpin the profession. Such studies within art therapy have included technologies that are available for measuring brain activity and that allow for movement inherent in artmaking (King and Kaimal, 2019) including quantitative electroencephalography (qEEG; Belkofer and Konopka, 2008; Belkofer et al., 2014; Kang et al., 2023; King et al., 2017; Kruk et al., 2014; Pénzes et al., 2023), functional magnetic resonance imaging (fMRI; Kang et al., 2023; Walker et al., 2018), and functional near-infrared spectroscopy (fNIRS; Kaimal et al., 2017). From these studies, evidence of the impact of specific art making processes, particularly drawing activities and clay work, has illuminated specific neural pathways of activation which invoke sensory, motor, emotional, perceptual, and executive functioning involved in association, analysis, regulation, and cognitive synthesis. Further comparative research has combined neuroimaging with other physiological measures to examine the impact of specific art materials on various autonomic, emotional, cognitive, and physiological systems (Haiblum-Itskovitch et al., 2018; Kaimal et al., 2016; Rankanen et al., 2022; Yang et al., 2021). Art making, as examined within art therapy neurobiological research, has also shown benefits for regulation, reducing stress, and improving mood, for example, which support the therapeutic evidence base for art therapy as a profession (Abbing et al., 2023; Beerse et al., 2019; Kaimal et al., 2016, 2017; Yang et al., 2021).

### 1.2 Social cognitive neuroscience research: racism, implicit bias, and implications for research practices

It has become imperative across healthcare, particularly mental healthcare, for research to examine the impact of social constructions of racial bias, prejudice, and discrimination throughout multiple levels of organized society (Banaji et al., 2021; Braveman et al., 2022; Berger and Miller, 2021; Berger and Sarnyai, 2015; Eckstrand et al., 2022; Kaiser Trujillo et al., 2022; Kubota, 2024). Social cognitive neuroscience research has been instrumental in uncovering underlying root causes of health disparities, specifically structural and institutional racism, among Black and other marginalized communities, and how the impact of racism and discrimination (i.e., implicit and explicit biases, and prejudice) shaped cognitive biases across all communities (Kaiser Trujillo et al., 2022; Webb et al., 2022b). Specifically, discrimination and explicit biases perpetuate increased environmental and interpersonal stressors leading to health conditions (e.g., trauma, depression, anxiety or psychotic disorders, obesity, cardiovascular disease), other metabolic irregularities, and impact neurocognitive development (Berger and Sarnyai, 2015; Hardeman et al., 2022; Rebello and Uban, 2023; Webb et al., 2022b). Various studies have elucidated neural structures involved in, and responses to, racial recognition, perception, and processing to further understand biases that underscore prejudices and implicit responses (Ito and Bartholow, 2009; Kubota et al., 2012; Rösler and Amodio, 2022). Neuroscience research has also explored the impact and measurement of implicit bias as evidenced by neural activity, hormone and neurotransmitter characteristics, along with other physiological measurements related to emotional and autonomic nervous system responses (Cassidy and Krendl, 2016; Morehouse and Banaji, 2024). Distinct differences at the neurological level have been delineated and then attributed to the effects of racism and discrimination underscoring the cognitive, emotional, and somatic impact of implicit and explicit biases (Kaiser Trujillo et al., 2022; Kubota et al., 2012; Rebello and Uban, 2023) leveled across marginalized and minoritized groups and communities (Berger and Sarnyai, 2015).

However, there has also been increased scrutiny of all aspects of healthcare research as Black, Indigenous, and other People of Color (BIPOC) communities have been poorly represented and depicted across healthcare data with a lack of relevant and beneficial studies generally, partly due to disparities in funding and the lack of funded research examining healthcare conditions and systems relevant to minoritized communities (Cené et al., 2023; Gilpin and Taffe, 2021; Hardeman et al., 2022; Orr et al., 2021). Researchers within social work, biological psychiatry, psychotherapy, cognitive neuroscience, and public health have contributed discourse to address research practices and attitudes that limit awareness, perpetuate exclusions, and that limit diversity of participant samples (Eckstrand et al., 2022; Green et al., 2022; Goings et al., 2023; Goldfarb and Brown, 2022; Kwasa et al., 2023; Orr et al., 2021; Paquin et al., 2019; Ricard et al., 2023).

#### 1.2.1 Understanding racism in neuroscience research

A closer look at cognitive neuroscience research related to implicit biases and structural racism has led to increasing awareness of structural racism maintained across cognitive neuroscience research design and subsequent critical analysis (Abiodun, 2019; Choy et al., 2022; Gilpin and Taffe, 2021; Louis et al., 2022; Parker and Ricard, 2022; Ricard et al., 2023; Webb et al., 2022a, 2022b). Notably, critical literature within neuroscience revealed illumination of issues of inequality across research design including the method, recruitment, exclusionary factors, technology, and funding practices (Gilpin and Taffe, 2021; Girolamo et al., 2022; Ricard et al., 2023; Rollins, 2021; Wassenaar and Van den Brand, 2005; Webb et al., 2022a, 2022b). Participant samples lacked diversity linked to insufficient recruitment considerations (Henrich et al., 2010; La Scala et al., 2023), publications rarely provided thorough descriptions of the samples or contextual information (Goldfarb and Brown, 2022; Kwasa et al., 2023), and there are marked disparities in both the funding allotted for minority health issues and awarded to researchers from racial and ethnic minority groups (Abiodun, 2019; Gilpin and Taffe, 2021). Additionally, aspects of reliability and validity of available neuroimaging technology (e.g., EEG, qEEG, fMRI, and fNIRS) have been scrutinized relative to hair and skin phenotypes which directly and indirectly limit inclusion and contribute to exclusionary practices (Choy et al., 2022; Couch et al., 2015; Green et al., 2022; Louis et al., 2022; Kwasa et al., 2023; Wassenaar and Van den Brand, 2005; Webb et al., 2022a, 2022b).

#### 1.2.2 Antiracist neuroscience research

Antiracism is the practice of confronting and dismantling racism through beliefs and practices that support equality across all ethnic groups previously marginalized through inequitable practices (Kendi, 2023; Paradies, 2016). Antiracist, equitable, and ethical clinical practice and research ideologies are facilitated through critical analysis of traditional practices and ideologies that foster structural racism, oppression, and exclusion (Rollins, 2021; Kaiser, 2017; Sajnani et al., 2017). The exposure of racist and exclusionary practices in neuroscience research design has led to practical and theoretical perspectives for quantitative, human subjects’ research, development of an antiracist framework, and a call for enhancements in neuroimaging technology (Choy et al., 2022; Goings et al., 2023; Goldfarb and Brown, 2022; Henrich et al., 2010; Parker and Ricard, 2022; Ricard et al., 2023; Rollins, 2021; Webb et al., 2022a, 2022b). Growing literature outlining antiracist ideology suggest critical reflexivity is required for White practitioners and researchers in the health professions to examine their authentic responses to concepts of inequality, oppression, marginalization, racism, biases, and discrimination to inform behaviors, attitudes, and perceptions (Etherington, 2017; Williams et al., 2022). Critical reflexivity requires the researcher to identify and examine their values and beliefs which shape both their intention to conduct research with specific aims and goals and their interpretation of the phenomena being studied (Bourke, 2014; Etherington, 2017). Additionally, it is imperative to clarify research questions to include a critical examination of who benefits from the research (Gilpin and Taffe, 2021). The aim of antiracist research is to enhance the lives of the community centered in the research by addressing concerns and problems identified by the community (Goings et al., 2023; La Scala et al., 2023). Researchers working from an antiracist framework will collaborate with the communities they intend to study, and involve diverse, interdisciplinary teams in their research efforts to ensure differing perspectives (Goings et al., 2023; La Scala et al., 2023).

#### 1.2.3 The imperative need for antiracist approaches in art therapy research

Within the field of art therapy, similar analysis of inequities and imbalances throughout the profession have elicited increasing critique and calls for greater examination of ambivalence about demographic representation of professionals and dominant narratives (Hadley, 2013; Kapitan et al., 2011; Sajnani et al., 2017). Social justice and advocacy discourse has existed in art therapy literature for decades, but has increased in recent years, in response to the growing divisiveness and hatred throughout America (Kuri, 2017; Talwar, 2017). A social climate toward dismantling systemic and structural racism within art therapy has grown more pressing (Potash, 2020). Antiracist discourse and literature within the art therapy profession appears to have emerged in 1999 (Cherry, 1999), and expanded after 2010 with growing emphasis on social justice, intersectionality, and ethically necessary antiracist practices to ensure applicability, relevancy, and effectiveness of art therapy practice and research (Hadley, 2013; Kaiser, 2017; Karcher, 2017; Kuri, 2017; Sajnani et al., 2017; Talwar, 2010; Talwar and Sajnani, 2022). Potash (2020) presented a charge for art therapists to become antiracist clinicians by developing an antiracist perspective, reexamining core art therapy concepts, and by pursuing systemic change. While neuroscience research continues to enhance the evidence-base of art therapy practice (Strang, 2024), research and practice standards could reflect antioppressive efforts to dismantle structural racism (Kaiser, 2017; Kuri, 2017). Aspects of power and privilege exist within the psychotherapeutic relationship between an art therapist and their client, while art making offers a mechanism to influence agency, empowerment, and to enhance equity (Kaiser, 2017; Karcher, 2017; Sajnani, 2012; Sajnani et al., 2017; Van Den Berg and Allen, 2022; Wright and Wright, 2022). Art therapists have unique traits of creativity, kindness, and curiosity (Braganza et al., 2023), as well as optimism to support adaptability and growth (Pénzes et al., 2018). These aspects combined with an emphasis toward social justice advocacy within a wide range of community-based systems are imperative to mitigate structural imbalance within those systems (Karcher, 2017). In fact, research has been emerging that the arts and application of aesthetic and creative processes may mitigate implicit biases (Garcia-Arch et al., 2020). However, art therapy research literature lacks acknowledgement of the discourse from cognitive neuroscience about racial bias and inequitable research.

Guided by the ethical principle of justice and in alignment with the American Psychological Association race and ethnicity guidelines in psychology (APA Task Force on Race and Ethnicity Guidelines in Psychology, 2019), this study aimed to conceptualize an antiracist perspective within art therapy neuroscience research and to contribute valuable insight necessary for systemic change.

## 2 Materials and methods

This qualitative pilot study incorporated semi-structured interviews to generate responses about problematic dominant ideologies embedded in neuroscience research and about antiracist practices in experimental art therapy neuroscience research. While a critical theory paradigm is implied in the current study which sought to conceptualize antiracist research practices, this pilot study was a preliminary attempt to examine perceptions, responses, and beliefs of a small group of experts in art therapy neuroscience research. The research question guiding the inquiry was: How do art therapy researchers interested in the integration of neuroscience and art therapy conceptualize antiracist research practices in neuroscience-focused art therapy research? Grounded theory analysis provided a structured, systematic, and rigorous approach to cultivate a new theory for advancing antiracist research efforts in art therapy. Specifically, the examination of perceptions, responses, and beliefs of a select, informed professional group of experts in art therapy neuroscience research contributed valuable possibilities to build a new theory about research practices from an antiracist lens (Leavy, 2017; Spencer et al., 2014). Approval of this study was obtained from the Lesley University Institutional Review Board (IRB).

### 2.1 Participants

For this exploratory pilot study, purposive sampling was appropriate to target the relatively small pool of potential participants who have conducted and published art therapy and neuroscience research. Inclusion criteria for this study specified that participants must be professional art therapists (e.g., completed graduate education in art therapy to possess the title of art therapist), who had notable experience developing, conducting, and publishing neuroimaging studies, and who were available for an interview between December 2023 and March 2024. The art therapists who were involved in the studies only needed to be listed as an author in the publications, not only the primary author. Exclusionary criteria included researchers who took part in neuroimaging research of artmaking processes who could not be professionally titled art therapists (e.g., cognitive neuroscience researchers, psychologists, neuroimaging technicians, biostatisticians), art therapists who conducted and published neuroimaging research prior to 2017, or other creative arts therapists conducting neuroimaging studies in other art modalities (e.g., music therapists studying impact of music). An in-depth search using research databases available through Lesley University library databases, including @LLSearch, JSTOR, Academic Search Premier, BioMed Central, EbscoHost, PubQuest Central, FLO library catalog, MEDLINE, and PsychInfo resulted in a list of eight relevant quantitative experimental studies published between 2017 and 2023 that included an art therapist and the use of neurobiological measurements to examine the impact of artmaking framed within art therapy principles (Cucca et al., 2021; Haiblum-Itskovitch et al., 2018; Kaimal et al., 2017; Kang et al., 2021; Kang et al., 2023; King et al., 2017; Pénzes et al., 2023; Rankanen et al., 2022). Invitations to participate were sent via authors’ emails to a list of 12 researchers and included a few questions for recipients’ responses to ensure they fit with inclusionary criteria and an attached informed consent form. The informed consent form explained the study, detailed the risks inherent in discussing issues of racism, and noted there could be no anonymity as the researcher would be directly engaging with them but described protections including deidentification to provide confidentiality. Following email interaction and verification of inclusionary criteria, informed consent was obtained from four participants (*N* = 4), and interviews were scheduled. The small sample was both reflective of the targeted sampling and was considered proper for a preliminary pilot study. There was no compensation offered for participation in the study.

As a pilot study, the intended number of participants was less than the typical number of participants needed for a full qualitative research study. The participants were female, middle-aged, doctorate-level art therapist researchers with varying cultural backgrounds. Each had more than 10 years of research experience connected to their own academic pursuits or as part of student academic research.

Three identified as White, and one identified as Asian American. One participant was born in the U.S., one was born in India, and the other two were born and resided in northern European countries (Finland and The Netherlands). The American-based participants identified also as administrators of art therapy graduate programs, one of the European-based participants identified also as a mental health scientist, and the other a clinical psychologist by education and training.

### 2.2 Researcher perspective

I am currently a doctoral student and have previously engaged in master’s level experimental research on art making processes and brain activity through qEEG (Kruk et al., 2014). Admittedly, qualitative research methods, while appropriate for the research design reflective of the research question, were less known outside of doctoral-level research education. I presented my positionality statement to frame the perspective and cultural viewpoint from which the impetus for the research and the research question was derived: “I am a middle-aged, White, female, English-speaking, able-bodied, American art therapist and doctoral student who has engaged in neuroscience research and I have elected to study, from a critical, postmodern perspective, how art therapy research practices have been perpetuating racism in neuroscience research and how a community of art therapy neuroscience researchers may conceptualize antiracist neuroscience research practices.” It is important to note that I was informed, prior to data collection and analysis, by the content of the articles which detailed concepts of racism and perspectives of antiracist research practices for neuroscience research from neuroscientists. Further, potential bias from preconceived notions or confirmation bias due to the influence from provided literature were considered as possible limitations.

### 2.3 Procedures

Upon reflection that the research question arose from a review of literature centered on illuminating imbalance and racism in neuroscience research, and that there is a lack of discourse on antiracist research practices among art therapy publications and journals, I made assumptions that concepts of racism in neuroscience research and discourse about antiracist neuroscience research would be new considerations among art therapy researchers. Therefore, taking inspiration from an antiracism exercise provided to undergraduate neuroscience students (Roth and Gavin, 2021), three recent, peer-reviewed articles relevant to the study topic were sent to the participants as an optional pre-interview reading activity. The articles provided a framework for the critical examination of neuroscience research and outlined inherent racial bias in current research practices (Gilpin and Taffe, 2021; Girolamo et al., 2022; Webb et al., 2022b). Three of the four participants reported they had reviewed the articles prior to their interview. For the participant who had not read the articles prior to the interview, a general overview of each article was verbally relayed to ensure they had sufficient information from the reading to elicit their responses. All participants reported they gained awareness about racist practices in neuroscience research and efforts to advance antiracist research.

#### 2.3.1 Data collection

In-depth, semi-structured interviews were conducted between December 2023 and March 2024, and each lasted from 40 to 60 min and were recorded via a confidential videoconferencing platform. Participants were reminded of their informed consent and right to withdraw from the study at any time. The interviews included open-ended, descriptive, and paradigmatic questions designed to examine previous experiences with neuroscience research, to elicit considerations about inequalities and limitations within research methods in neuroscience research design, to respond to the articles that were sent, and to explore conceptualization of requirements for antiracist art therapy neuroscience research approaches. The interview questions were developed carefully, so as not to lead discourse toward consensus, and an interview guide provided general structure for each interview but allowed for variation depending on different perspectives elicited from each participant (see Supplementary Item 1). For example, following the first interview, a question about implicit biases was added for the subsequent interviews. Participants were also invited to consider or provide their positionality statements to acknowledge their own intersectionality and cultural viewpoints relevant to, and surrounding development of their research (La Scala et al., 2023). Suggestions for advancing the dialogue about actionable antiracist practices within art therapy research were solicited. The interviews were transcribed carefully from the videoconferencing platform recordings which supported accuracy. A numbered identifier was assigned to each of the transcriptions which were saved as files to a password-protected file on the researcher’s computer. Prior to coding, the transcriptions were sent to the participants for member-checking to ensure accuracy and to clarify or add any sentiments. Three participants provided clarification or acknowledgment of the accuracy of their transcript.

#### 2.3.2 Data analysis

The principal researcher conducted all aspects of data analysis to include transcription, coding, and refinement of results. Transcription, incorporation of participant’s clarification from member-checking, and iterative reading allowed for in-depth familiarization with the material (Silver and Lewins, 2014). Separately, a reflexive research journal was used to bracket the researcher’s beliefs and notions gathered from the literature review (Gilgun, 2014). Analytic memos were included in the journal to capture notes about main salient points, and areas of consistent reflections from the participants across interviews (Saldaña, 2021). Because the researcher was aware her experience with art therapy and neuroscience research was aligned in many ways with the participants,’ reflexive journaling offered a container for subjective responses, insights, and allowed connected points to be documented during coding rounds (Trent and Cho, 2014). Using coding strategies in line with grounded theory analysis, the first two rounds of coding were conducted using iterative, open coding strategies, to include process and *in vivo* coding, and analytic memos (Saldaña, 2014). An inductive analysis method was applied while engaging with the content to begin to identify selected words or phrases as codes (Saldaña, 2021). Throughout the in-depth review process of each transcript, patterns of similar sentiments were color-coded across the transcripts which resulted in a focused coding strategy to provide an initial framework for organizing patterns of codes into core categories (Saldaña, 2021). There were 701 codes organized into a code structure of 10 core thematic concepts that emerged consistently across transcripts and provided the initial code structure. For further organization, the transcripts and manually developed code structure were imported into computer assisted qualitative analysis software, MAXQDA (VERBI Software, 2021). The use of MAXQDA provided development of visual coding and mapping structures, paraphrasing, summary tables, a codebook, and other analytical tools instrumental in saturation of the data and for clarification to articulate the themes evolved from the initial emerging categories (VERBI Software, 2021). Codes identified within the 10 core categories of the code system were collapsed across transcripts in subsequent rounds of organizing codes, further refining the data and leading to deeper interpretation (Saldaña, 2021). Paraphrasing was used to discern patterns and categories of codes more clearly across transcripts and the researcher’s journal memos which resulted in three overarching core categories, *Shared Beliefs and Values, Barriers and Challenges, and Requirements and Responsibilities for Antiracist Research*, that contained seven main themes and five subthemes. See Table 1 for further details and verbatim quotes.

**Table 1:** Anti-racist research in art therapy neuroscience research: categories, themes, & subthemes.

Core categories	Main themes & subthemes	Sample verbatim quotes
Shared Beliefs & Values	1. Neuroscience-informed Perspective of Art Therapy 2. Neuroscientific Research Can Strengthen Art Therapy Theories 3. Infusion of Antiracism and Neuroscience into Art Therapy Begins with Education	“…led me to cognitive neuroscience, naturally, because art therapy is such a highly cognitive and creative approach even though it does use all of our senses whether we realize it or not.” (Participant 1, [P1]) “…we taught students to do this neuroscientific research where we were curious about what happens in the brain when you use different kinds of art materials…” (Participant 2, [P2]) “…so the competencies for art therapy education have shifted and evolved over the years to now include more specific neuroscience content.” (P1)
Barriers & Challenges	4. Potential Credibility and Legitimacy Concerns for Art Therapy 5. Difficult Conversations about Disparities in Awareness, Diversity, & Resources	“I think [art therapists] have a perception problem even though its increasingly showing up in pop culture and movies and all of that, we do have a perception problem that we need to continue to chip away at…” (Participant 3, [P3]) “We have a long way to go in neuroimaging research with our participant samples that include diversity.” (P1) “Unfortunately, like a lot of the people who think about these things… are not the ones who should be thinking about it.” (P3)
Requirements & Responsibilities for Anti-racist Research	6. Due Diligence to Build Accountability & Legitimacy: Invite Awareness to Shift Perceptions Critical Reflexivity is Critical Learning Shared Language 7. Inclusion of Diversity in Art Therapy Research Collaborative & Interdisciplinary Approaches Decision-Making in Research Design	“I was glad of learning that the technology aspect [of limitations in research] were related to the hair type and the skin color… …and that if it’s possible to develop solutions to overcome those…” (Participant 4, [P4]) “We did not know, so I think awareness and knowledge they go hand in hand, of course….” (P2) “…somehow, I think, that then [positionality] could explain something of the… kind of like bias in the, potential bias, in the research.” (P4) “a lot of people are throwing around a lot of different words about things that need to happen and a lot of people are saying we need to have a similar language, but there is not a universal taxonomy yet…” (P1) “…you have to include this diversity, and I think one way of doing this is to go into the community…” (P2) “I think we have to recruit champions of art therapy from neuroscience. And recruit them as allies for our work.” (P3) “…this measurements aspect, and the technology aspects, that… has to be taken into account…” (P4)

Two opportunities for member-checking were offered to enhance credibility. The transcripts and the compiled results were sent to participants with invitations to provide correction, clarification, and additional commentary. Three participants provided comments and corrections after reviewing their transcripts. The updated transcripts were used in the analysis. Following the review of the results, two participants responded with areas of agreement, further commentary, and clarification.

## 3 Findings

Regarding participant selection, it was notable there were very few male art therapy neuroscience researchers identified, and none whom had published within the timeframe noted. Four participants all identified as female with little racial variation across the participant sample. There was one participant of Asian origin; however, nationality was the main difference across participants. The U.S.-based participants were astutely aware of wide variety within demographics relevant to their locations while the European participants noted a lack of racial diversity within their countries of origin. Three of the participants came from origins in which English was not their first or native language, and though geographical origins were different, the concepts and fundamental philosophies conveyed by the participants as art therapists, researchers, and educators were intriguingly concordant. Because of the small sample size, the findings, while compelling, are preliminary and do not support definitive conclusions.

### 3.1 Core categories, thematic concepts, and subthemes

The three core categories, *Shared Beliefs and Values, Barriers and Challenges,* and *Requirements and Responsibility for Antiracist Research,* reflected possible convergence of views and research interests, perceptions about challenges facing art therapy and neuroscience research and limitations to overcome, and suggestions for antiracist research relevant to art therapy neuroscience research. The seven main thematic concepts nestled amidst their respective categories (Table 1) further illuminated strongly conveyed points within the core categories, and themes within *Requirements and Responsibility for Antiracist Research,* contained subthemes.

#### 3.1.1 Shared beliefs and values

Each participant had been invited to share their positionality and how their life experiences had led them to both art therapy and their ongoing interest in neuroscience. All participants were professors within graduate-level art therapy training programs, had completed doctoral-level research, and described their natural inclination toward studying the brain in concert with their understanding of their own art therapy practice. Even across different continents the participants presented several seemingly similar underlying beliefs reflective in three main thematic concepts: *neuroscience-informed perspective of art therapy, neuroscientific research can strengthen art therapy theories, and infusion of antiracism and neuroscience into art therapy begins with education*.

##### 3.1.1.1 Neuroscience-informed perspective of art therapy

These participants shared longstanding curiosities about neuroscientific correlations of art making; therefore, their application of art therapy theory and methods into practice and research was inherently, and primarily, understood from a neuroscientific framework. Participant 1 (P1) noted, “over time that led me to cognitive neuroscience, naturally, because art therapy is such a highly cognitive and creative approach even though it does use all of our senses whether we realize it or not.” An understanding of neurobiological processes during art making was considered relevant because “there’s something about the expressive process and what that feels like […] like people feeling like improved mood […] reduced inflammation, relaxation in the response, feelings of, like positive emotions in particular reward perception” (Participant 3, [P3]). Connections between artmaking and neural processes were highlighted to underlie principles of art therapy, or “tenets: non-verbal, symbolic communication, use of creative expression that perpetuates healing and beneficial outcomes, that engages different areas of the brain and body, the materials and methods that we thoughtfully use and apply in our work” (P1). Research on art making within art therapy and subsequent impact on mind and body inducing therapeutic effect was a priority, as Participant 2 (P2) noted, “we taught students to do this neuroscientific research where we were curious about what happens in the brain when you use different kind of art materials and when you use different kind of instructions.” Collectively, neuroscience principles guided their understanding about mechanics of mind and body involved in therapeutic use of art making, and mechanisms of change from art therapy.

##### 3.1.1.2 Neuroscientific research can strengthen art therapy theories

Their shared beliefs and interests in the intersection of neuroscience and art therapy underscored research pursuits to bolster the field of art therapy practice and build knowledge of neurological processes involved in art therapy processes. P2 provided, “I’m always doing research because I want to know; I want to gain knowledge,” and elaborated, “I am a big proponent of practice- based research, just because I want to connect research to practice and education.” P1 reflected on various experiences in her career “involved in the realm of what I call systematic integration of neuroscience and art therapy for over a decade,” and training which has “helped me understand more about how to navigate some of these chasms that exist between the more cognitive, basic neuroscience research and what it is that we do in art therapy theory and practice.” Participants described their drive to examine artmaking for benefits and applications, to understand the impact of art materials and processes, and to elucidate the mechanisms of therapeutic effect to move beyond theoretical knowledge toward an evidence base. While research in both art therapy and neuroscience fields has grown, participants implied both fields primarily operate from theoretical knowledge and require a range of research efforts to deepen comprehension within and across disciplines. Realistically, P1 highlighted the need for expanded research efforts in art therapy regarding the underlying mechanisms of art making because “we know that making art is really different than rote motor movement, but we cannot make that leap to saying that it is without taking […] teeny-tiny baby steps along the way” and, therefore, “some of that really controlled experimental design is necessary” to enhance research, practice, and education standards. P2 pointed out how “neuroscience research is actually more fundamental, less practice-based” and stressed the “importance of being aware of how research findings find their way through publications to practitioners and educators” because that information becomes incorporated into practice and perpetuated through education. These participants similarly believe neuroscientific art therapy research builds support for development and strengthening of theories, and how basic, empirical research findings may lead to applied research to elicit evidence to support advancement of the profession.

##### 3.1.1.3 Infusion of antiracism and neuroscience into art therapy begins with education

From their perspectives as both researchers and educators, participants considered how educational criteria in art therapy could evolve to incorporate both a neurobiological perspective into the theoretical underpinnings of art therapy and knowledge about inequalities in human subjects’ research design. P1 reflected on her experiences “in program development and curriculum development and looking at how neuroscience underpins our theory in practices,” and noted that “the competencies for art therapy education have shifted and evolved over the years to now include more specific neuroscience content.” Yet, it was also acknowledged more work is needed to augment professional education standards across the profession because “there seems to be a gap in learning […] and in knowledge acquisition because many educators in our profession might not have a good foundation of neuroscience” (P1). In concert with an infusion of neuroscience principles, an antiracist lens was considered essential to future research standards within art therapy. There was a sense of revelation and then duty to act as the participants responded to new, “really eye-opening” information about the inequalities and exclusionary practices identified amongst neuroscience studies, which led to a strong realization that “we have to be more aware as researchers how we impact practice, education with our research findings” (P2). Summarily, P1 provided that for a commitment to development of antiracist research “this kind of content is necessary for graduate art therapy research coursework […] So, this needs to be embedded, similar to neuroscience, it needs to be more firmly embedded in our education curricula.” In agreement, P2 reflected “To create this awareness [about antiracist research] or to enhance this awareness? Well, I’m an educator, so I think, start there.” Incorporating antiracism and neuroscience principles into art therapy education to expand awareness and promote antiracist, ethical, and equitable practices was a strong suggestion reflective of participants’ viewpoints that such knowledge can benefit the growth of the field in general.

#### 3.1.2 Barriers and challenges

Participants acknowledged new awareness about the issues prompting consideration for antiracist art therapy research, and identified perceived barriers in the way of fully embracing and incorporating this perceptual shift. It was easy for participants to name various challenges to transform approaches within art therapy neuroscience research because an evolution toward more equitable antiracist research approaches would require critical examination and conceptualization of credible research methods. These art therapy researchers invested their time to consider a comprehensive conceptualization about what such transformation would entail. There were questions raised about the practical implementation of changes in research practices along with some doubts related to lack of certain resources, and concerns about the reputation of the art therapy profession in general. These concerns about the perception of art therapy stemmed from first-hand experiences conducting and contributing research generated within the field of art therapy to the wider mental health behavioral healthcare systems. Experiences with divisiveness, perceived scientific hierarchy, and clashes within and outside of the profession ushered the emergence of the theme of *potential credibility and legitimacy concerns for art therapy.* Further, there was discussion about consideration for diversity in human subjects’ research, concerns about the availability of resources to include neuroimaging technologies, and overarching concerns a paradigm shift would face barriers reflected in the second overarching theme *difficult conversations about disparities in awareness, diversity, and resources*.

##### 3.1.2.1 Potential credibility and legitimacy concerns for art therapy

Participants shared their first-hand experiences and challenges as art therapists, researchers, and educators who have witnessed and been challenged by the absence of widespread familiarity with art therapy as a healing profession, despite decades of work and research conducted internationally. From her location in northern Europe, P4 described “that there is no art therapy department or program or professor or anything” and her education and work was conducted “under the department of art and under the discipline of art education […] and then [her] supervisor was a psychologist who was specialized in psychology of art.” There was a feeling of disappointment generally related to the lack of awareness about what the profession of art therapy is which contributed to a sense of pessimism and frustration, as P3 noted, “So there’s a real fatigue to all of this […] and I do not want to undermine that, there’s a real fatigue to explaining [about art therapy].”

Assumptions, lack of understanding, or lack of awareness about the profession created disconnection from other fields and inaccurate and judgmental perceptions of the veracity of art therapists as researchers and practitioners. There was awareness about a perceived hierarchy across disciplines and about how art therapy research is regarded, “so we are lower on the [perceived hierarchy], when it comes to establishing [art therapy] from a scientific basis […] and neuroaesthetics is much higher up because historically the research is experimental and takes place in the lab” (P1). Notably, P3 provided clear examples of judgmental perception from the reaction of one “collaborator saying, ‘Well next time, let us make sure we bring a psychologist on board, so that, you know, we are seen as more credible,’” and from a discussion with a neuroscience colleague that art therapy is considered “mumbo-jumbo” as he described how his peers “make fun of him for his interest in art therapy.” This perception problem was viewed as a responsibility for the field of art therapy to acknowledge and address. Issues of credibility and legitimacy of art therapy were perceived to stem from both a lack of operationalized definitions and scientific evidence because art therapy principles remain largely theoretical. Participants based in the U.S. framed issues of division within the art therapy field, “because [art therapists] end up fighting about what our definition is,” because “the variations of what [art therapists] do are immense, and it’s really complicated what we do and it’s hard to define” (P1). Connecting to that point, P3 reflected, “We do have a harder hill to climb, though, as art therapists, I think,” describing how a lack of a scientific foundation contributed possible barriers related to the issue of credibility about “art therapy as a profession, being assumed to be like, I do not know, hippie-dippie something that people do not understand. That […] we do not understand the sciences.” Development of the scientific basis of art therapy, according to these participants, may facilitate a stronger definition of art therapy through a neuro-informed theory of the therapeutic mechanisms.

##### 3.1.2.2 Difficult conversations about disparities in awareness, diversity, and resources

Participants considered problems with a potential lack of widespread awareness about issues raised in neuroscience literature about inequalities, the lack of diversity as an aspect of exclusion in research, and a general lack of necessary resources. Frustrations about limited resources, primarily funding and opportunity, were raised as P3 noted, “anything that requires a resource, like equipment, there are costs associated with it” which is a concern facing art therapy research efforts since “there are expenses involved which already kind of set up a disparity.” The use of neuroimaging was a desired focus for participants’ research designs, and they overcame technical barriers through collaborative work with technicians and other scientists, “but we never, ever, ever thought about the influence of, yeah, your nationality of your hair, or your skin color might have on our measures. […] Why did we never, ever thought about this?” (P2). Considering the decades of published neuroscience research, there were questions about why issues related to inequalities in neuroscience research design have only recently sparked movements toward antiracism. Participants contemplated the lack of awareness, discourse, and activism toward equitable research standards within art therapy, as P1 identified, “it’s distressing that some of our thought leaders […] do not seem to have knowledge about [inequalities in neuroscience research] or choose to look the other way,” rather than conducting critical analysis of research practices. As P3 explained “like in a lot of antiracist research and in a lot of […] intersectionality research, the ones who should be thinking about this are blissfully oblivious.”

Awareness about the importance of diversity in human subjects’ research brought up considerations about the research setting and location where studies were conducted as some countries lack diversity generally, so “the recruitment it’s really problematic” (P4) in northern Europe, for instance. In general, the analysis that neuroscience research has predominantly focused on homogenous, mostly White, participant samples has fueled new skepticism as P4 noted, “so much like generalizing White, University students and telling […] that ‘This is the human mind’.” Therefore, there was a question about how the lack of diversity may impede generalization of research findings across populations or might influence a skewed formation of knowledge of human cognition, “recognizing that a lot of the data that we have, and what we are purporting to be truth, has been founded on very specific patient samples that do not include a diverse scope” (P1). Participants were aware there is a certain expectation for rigor within scientific experimentation to meet acceptable standards. Biases, implicit and explicit, were identified both regarding research (e.g., confirmation bias) and as inherent to the researcher’s positionality, but positionality is not typically included in quantitative scientific research “because […] that is also like the difference between qualitative and quantitative research […] if you do quantitative research, yeah, it has to be objective” (P4). However, participants also conveyed that cultural aspects affecting researcher’s perceptions and interpretations might present other confounds if not considered.

#### 3.1.3 Requirements and responsibility for antiracist research

These professional art therapist researchers shared honest reflections, hopeful sentiments, revelations on how to enhance antiracist art therapy neuroscience research practices, and appreciation for the opportunity to participate in elevating the discourse. While this study elicited concerns and questions, participants also considered many strategies beneficial for increasing a socially-just stance in art therapy human subjects’ research. There was recognition of inherent traits and characteristics of the art therapist identity, including adaptability, flexibility, and creativity, which may be essential to facilitate incorporation of antiracism into art therapy research, “and that’s what [art therapists are] best at […] we are very creative; we have the ability to think outside-of-the-box” (P1). Considerations about incorporating an antiracist framework into research practices were predicated on shared beliefs that neuroscientific research can strengthen art therapy and art therapists act in ways that support social justice. Two themes seemed to emerge somewhat like principles to be incorporated into art therapy research standards. The theme *due diligence builds accountability and legitimacy* contained subthemes *invite awareness to shift perceptions, critical reflexivity is critical,* and *learning shared language*. The other theme in this category, *inclusion of diversity in art therapy neuroscience research,* contained the subthemes *collaborative & interdisciplinary approaches* and *decision-making in research design*. The interconnected subthemes intimated potential steps forward toward change.

##### 3.1.3.1 Due diligence to build accountability and legitimacy

Reflective of challenges they had faced in pursuing their research interests, participants noted their own due diligence to build accountability and to convey legitimacy which underscored suggestions for the work required to understand and undertake a movement toward antiracist research. This includes incorporating deliberate efforts in education, research, and practice to dismantle oppression and enhance equitable treatment that are “trying to get towards a greater justice, and so […] I have a responsibility to be an active part of that, just as much as I do to have a critical eye and a mindset and do my homework” (P1). Education in neuroscience and antiracist research allows art therapy researchers to expand their awareness, knowledge, and to be able to communicate effectively using a shared language in necessary interdisciplinary partnerships.

These researchers acknowledged their positionality from an art therapist’s framework and expertise, and emphasized the diligence required to conduct neuroscientific art therapy research that reflected ethics and equity. Diligence and transparency in research and publication were considered important to raise awareness about socio-cultural contexts of participant samples because potentially “this lack of evidence also affects practice and education, so it is important not only to be aware of this but to actively report these variables” (P2). Participants identified the importance of reflexivity and critical self-reflection for art therapy researchers to consider how their perspectives, cultural aspects, and experiences in practice and research can influence research design and outcomes. Further, for an informed perspective reflective of an antiracist paradigm honesty and acceptance help to identify and accept limitations and blind spots, or “what we do not know that we do not know” (P1). Reflecting on provided literature that promoted emphasis on antiracist practices, participants elaborated on possible responsibilities and due diligence of art therapy researchers to enhance personal and professional accountability through ongoing learning, awareness, and transparency, as noted in the subthemes *invite awareness to shift perceptions, critical reflexivity is critical,* and *learning shared language*.

###### 3.1.3.1.1 Invite awareness to shift perceptions

Participants described the lack of awareness as a considerable challenge to incorporation of an antiracist framework in art therapy research. They considered the easiest and most significant first step may be to invite awareness about the racism and inequality in historical neuroscience research to inspire perceptual shifts toward equitable research practices. Antiracist research practices can be promoted through professional discourse as P4 noted, “like knowledge exchange […] in conferences, having like roundtable discussions or […] like somehow lifting the subject [antiracist design within art therapy and neuroscience research] up” to increase awareness and inspire transformation. P2 identified, “Yeah, the word that pops up is knowledge, just knowing. We did not know, so I think awareness and knowledge they go hand in hand, of course.” This sentiment was echoed across interviews that to navigate incorporation of new knowledge and to enhance equitable research design “we all need to have greater awareness” (P1). Therefore, an expansion of this specific discourse through intentional avenues created to amplify the conversation about efforts to address the inequalities in neuroscience research was touted as an essential activity. An invitation to participate in difficult conversations to raise awareness may be required as noted by P3 because “people want to do the right thing, so when things are pointed out in a way that invites awareness” transformation and perceptual shifts are more likely to occur, and more likely because “sometimes people recognize and change behaviors when it’s not yelled at them in the face.” Participants shared feelings of gratitude and expanded awareness in response to their participation including opportunities to review provided articles and participate in interviews to contribute to change, “well I was really glad because I had not been aware,” (P4) and relief that the publications about inequalities within neuroscience research were circulating.

###### 3.1.3.1.2 Critical reflexivity is critical

Aligned with increased awareness, critical reflexivity and critical analysis of current processes and standards were promoted as potentially vital practices to incorporate into an antiracist framework. Development of statements of positionality were considered key for identifying potential influences as P4 identified that a researcher’s “specific cultural background that brings, like, certain kind of […] biases, […] to how we are conducting research, how we are doing therapy.” P3 considered how including positionality to reflect aspects of cultural influences and acknowledgement of privilege “is huge” for art therapy researchers. Several suggestions for critical self-reflection were posed across interviews, including questions considering “the culture we live in, the culture we are, kind of like, habituated to […] how is that affecting, and how do we take that into account, or even are aware of the potential effect?” Self-reflective questions related to researchers’ awareness of their own limited scope and their exposure to working with difference are very important for an antiracist framework because, as P3 raised, “we tend to be surrounded by people who are like us” which limits perspectives and beliefs. Honesty drove participants’ admissions about how a discussion about antiracist research practices and positionality required consideration, examination, and acknowledgement of perspectives that are “limited in my initial perceptions and scope,” (P1) and that “I have a total different reference and […] I try to be aware of this,” (P2) and “I think my, my perspective is really narrow or […] not experienced” (P4). This is not unlike certain trends in cultural humility and cultural competencies to identify implicit biases, but as noted by P3, “there’s not enough work done on, well, once you discover your bias, what do you do about that.” Acknowledgment of the unique qualities of the art therapy profession, art therapists (e.g., adaptive, flexible, creative, transcends culture and context), and the specific interest in neuroscience research inspired thoughts about implicit bias research initiatives. It was suggested art therapy neuroscience research is poised, considering these unique benefits and the “tenets” underlying art therapy processes, “to study implicit bias, to study unconscious processing” and “to capture information about bias and about […] race, about perception […] bias both implicit and realized” which may position art therapy neuroscience research at an important intersection of neuroscience, neuroaesthetics, arts, antiracism, and social justice (P1).

###### 3.1.3.1.3 Learning shared language

Reflective of their shared beliefs about the due diligence needed to enhance accountability and interdisciplinary knowledge, participants suggested art therapists interested in such scientific research have a responsibility to learn neuroscience principles, terminology, and technology to communicate effectively and to facilitate interdisciplinary collaborations involved in art therapy neuroscience research. As part of her own pursuit to conduct EEG research P1 reflected, “I’m a big advocate for contemporary neuroimaging and mobile imaging and technology to advance our work, so I’ve done my due diligence to learn about what that means.” For art therapists conducting neuroscientific research, “we have to be humble in terms of learning more of the language of neuroscience, while also knowing that there’s things [art therapists] know from our clinical practice […] our artistic practice, and our research practice that nobody else knows” (P3). Working with knowledgeable interdisciplinary partners (e.g., neuroimaging technicians, cognitive neuroscientists, and researchers in related disciplines of psychology or art education and design) has offered participants a broadened perspective on the benefits to and requirements for working together. Learning a similar language was noted to be vital to successful, diverse collaborations. Simply put, “we have to know the language of neuroscientists as well, so if we want them to respect us, we should respect their work” (P3). However, there is work to be done to bring about greater understanding within and across disciplines at the intersections of neuroscience, the arts, and creative arts therapies, because “the definitions are different depending on who it is that you talk to,” and “there is not a universal taxonomy yet, that allows us to traverse the different disciplines and definitions of […] core domains of which we are all speaking about” (P1). The language that we rely on to communicate our interests in building knowledge through research, and how we translate research findings into practice and education standards become paramount to shared understanding.

##### 3.1.3.2 Inclusion of diversity in art therapy research

Participants conceptualized a shift toward equitable and antiracist design by increasing diversity and through collaborative interdisciplinary approaches. Prioritizing diversity throughout art therapy neuroscientific research was identified by all participants as a potential key to infuse antiracism across education, research methodology, and dissemination of antiracist research. Participants noted it is important to engage in actions which may shift perceptions to ensure accountability toward an antiracist framework, for instance, by asking art therapy professionals to “intentionally seek out difference” to enhance inclusion within research and practice (P3). To deliberately seek out diversity to align with antiracist perspectives, participants reconsidered traditional recruitment methods that tend to focus on accessing university students and contemplated different approaches to engage potential participants. P2 suggested, “I think one way of doing this is to go into the community, and include not just students of the university but also people around the block; people from […] different nationalities and try to include them.” For equitable research practices, it was noted that researchers should also consider who benefits from the research and how research findings might be best translated into practice. Specific to art therapy neuroscience antiracist research practices, P1 reflected on the unique benefits of the practice of art therapy “because our work is just naturally very rooted in the community and because of the value of the arts to transcend cultural identities.” Acknowledgement of the researchers’ positionality highlighted the responsibility to remain mindful of limitations of our own perspectives and to consider equitable, ethical, and respectful approaches to recruitment, as P2 continued, “I think it would be great to, before you go out sampling, you talk to this minority groups or representatives of these groups to get a feel of how can we communicate.” A commitment to increase diversity across professional experiences, education, and all aspects of research design to include collaborations and participant samples was considered part of antiracist research practices because, “just by being present, just by working with someone who’s different from you, collaborating with someone from, different from you, presenting together, writing together, those will shift your perceptions” (P3). Therefore, future antiracist art therapy neuroscience research should consider expanding diversity throughout all aspects of the research process as reflected in the subthemes *collaborative and interdisciplinary approaches* and *decision making in research design*.

###### 3.1.3.2.1 Collaborative and interdisciplinary approaches

Participants discussed the importance of diverse and enlightening research collaborations which were intimated to be vital for art therapy neuroscience research. These researchers highlighted experiences with neuroscientists, engineers, neuroimaging technicians, statisticians, pharmacists, art educators, and designers who took part in research examining various questions about the impact of art making. Emphasis was placed on researcher identity amongst an interdisciplinary research team, as P4 observed she “was the only art therapist” who collaborated with other researchers on a neuroscientific research design focused on drawing tasks with aspects relevant to each distinct field. The interpretation of neurobiological responses to art making was dependent on each researchers’ framework, drawn from their knowledge and expertise, and represented how they conceptualized findings applicable to their respective fields. P3 illustrated the necessity to apply an art therapist lens while contemplating the results of neuroimaging research when discussing results with her cognitive neuroscientist partner, “I was like ‘I do not know, you tell me you are the neuroscientist!’ He was ‘No, but you are the art therapist, how does activation of this pathway affect something in your world?’” Expanded awareness and purposeful collaboration with knowledgeable partners may lead to the incorporation of new, diverse perspectives and knowledge, and unique insights to enhance antiracist practices in research.

There were sentiments expressed about the inclusion of contributions from transdisciplinary partners, and particularly seeking out knowledgeable partners. Specifically, for beneficial collaborative partnerships, “we have to recruit champions of art therapy from neuroscience and recruit them as allies for our work” (P3). P2 pondered an important consideration, “How can you reach out to other perspectives that not only enhanced the quality of your research, in terms of methodology, but also in terms of dissemination afterwards, towards community, toward practitioners and students?” As P1 noted, “the majority of my work has been in […] collaborative initiatives to bring together different disciplines to determine the best ways of going about learning more about what art therapy and neuroscience is and can be.” They contemplated accessing contributions from a wider range of perspectives along with collaborators who are already seeking to enhance inclusion and diversity in line with an antiracist paradigm in research.

###### 3.1.3.2.2 Decision-making in research design

The juxtaposition of antiracist practices that enhance diversity with traditional neuroscientific research practices elicited contemplation from the participants about possible challenges considering the history of neuroscience research practices, (e.g., exclusionary, homogenous and predominantly White participant samples, absence of subjective data, and lack of contextual descriptions of the sample). Participants considered basic requirements for art therapy and neuroscience research, for example “if you are conducting research in cognitive neuroscience and neuroaesthetics, then you need a neural variable and that’s part of experimentation” (P1). Further, art therapy research designed to explore the mind/body mechanisms of art making needs to include examination of the subjective experience to enhance meaningfulness, indicating the necessity of mixed methods approaches. Critical reflection of prior research literature publications inspired critiques of common publication practices in quantitative research, (e.g., providing limited description of participant samples and reliance on neuroimaging technologies which potentially led to exclusionary practices), and “that’s important to address, so that there’s more transparency about who was in your sample” (P2). To shift toward change within research practices, diversity should be incorporated throughout all decision-making in development of the research design, for instance, “you have to include this diversity,” and “to make sure […] your sample is diverse so you can generalize your findings to the whole population” (P2). P4 admitted “it’s also really complicated to, […] to try to map all these different [cultural and contextual] aspects [of the participant sample]” to ensure equality and balance in all aspects of research design and methods. Similar mindfulness including critical awareness about art conditions selected for experimentation was also an important point made for art therapy research in general, “being aware that these are culturally influenced” (P2). Regarding available neuroimaging technologies for conducting research with diverse sample populations, there was some curiosity noted by P2 while considering how to apply “a more multicultural perspective in research design,” that “with EEG, I would not expect any different measures from an African, or a Native American, or a Caucasian participant, but to know this helps you to design your research in a different way” (P2). This was noted to be important to consider further as P4 mused about awareness that “the technology aspect [of inequality in neuroscience research] were related to the hair type and the skin color. …And that if it’s possible to develop solutions to overcome those… that we should develop more of these kinds of measures.” As literature on antiracist research has expanded, P1 noted a snowball effect for advancements in technologies and research design because “tech companies that are building this equipment are paying more attention to the academic researchers… putting out the information about the importance of paying attention to [inconsistencies in available technology].”

In summary, preliminary results indicated these art therapy researchers share viewpoints about the importance of neuroscientific research for advancement of the field, considered possible challenges for such research in general, and potential actions to advance antiracist research practices. Participants raised points about increasing awareness of critical analysis of research practices, deliberate decision-making in the development of research design, possible benefits from incorporating diversity throughout research teams and across participant samples, and intentional collaborations with knowledgeable interdisciplinary partners. By evoking increased awareness and new knowledge for advancing and infusing antiracist research initiatives, art therapy education and research practices may align with current, and widely regarded, neuroscience research to potentially remedy noted disparities.

## 4 Discussion

With consideration of aspects of traditional neurobiological research that have come under scrutiny (Gilpin and Taffe, 2021; Girolamo et al., 2022; La Scala et al., 2023; Ricard et al., 2023; Webb et al., 2022a, 2022b), this study aimed to generate conceptualization of equitable antiracist research practices amongst a small group of art therapy neuroscience researchers. For these participants with multifaceted and intersectional identities, equitable and ethical research practices were considered important as shown by their responsiveness toward antiracism in neuroscience research. From this preliminary inquiry with art therapy researchers, discussions about inequities within neuroscientific research from an art therapist’s lens suggested promising outcomes which might promote innovation in antiracist research practices and design to expand the application, viability, and evidence base of the practice. Art therapy may inherently equalize communication and transcend differences across communities and throughout healthcare as art making provides a vehicle for communication of different perspectives on a level playing field (Griffith and Semlow, 2020; Hoshino, 2016). Additionally, incorporation of antiracist research practices into art therapy neurobiological research supports and reflects the values statement issued by the American Art Therapy Association (2017) including a “commitment to inclusivity” (para. 4), to “uphold social justice” (para. 7), and to “maintain awareness of the social and environmental consequences of human actions on communities, ecosystems, and associations, and strive to advance a sustainable and just society” (para. 8).

### 4.1 Core categories and connections

Notably, the themes within the first core category of *shared beliefs and values* reflected apparent alignment and synergy of viewpoints that drove participants’ research interests and practices. The veracity and viability of art therapy as a profession presumably requires diligence to succinctly define what art therapy is, to set the parameters for effective standards of treatment, and to operationalize art therapy treatment across mental health treatment for all ranges of people, diagnoses, and issues (Leslie et al., 2023; Shukla et al., 2022). There is a belief within the art therapy profession, across nationalities, in the obligation to produce research that shapes the field and “its capacity to influence policy and practice within the wider health care community” to “remain relevant, contemporary, and ethically grounded” (Carr and McDonald, 2019, pg. 54). These participants highlighted their belief in the drive toward a scientific basis of art therapy to dispel misperceptions, and to position this health profession as credible and legitimate amongst the larger domain of health professions. It was further considered important for art therapy research efforts to align with current cognitive neuroscience research efforts to address health equity disparities (Rebello and Uban, 2023; Webb et al., 2022b), including responsible consideration of participant demographics (Goldfarb and Brown, 2022) and increased awareness of available technology (Penner et al., 2023; Webb et al., 2022a), to reflect the “shared pursuit of antiracism” (Van Den Berg and Allen, 2022, p. 4) by naming antiracist art therapy research practices.

Similar to critical analysis within neuroscience research (Gilpin and Taffe, 2021; Parker and Ricard, 2022), a sense of a stark reality shone through the themes within the second core category, *barriers and challenges*, that reflected concerns for art therapy professionals, educators, students, and researchers to consider. Potential considerations include the lack of consistent definitions integral to art therapy practice, the need for review of education curriculum to reflect current research initiatives, and availability of equitable technologies. It may be important for conversations to occur within professional arts therapies organizations about the need for critical analysis of research methods, funding, and reverence and acknowledgement from interdisciplinary partners (e.g., cognitive neuroscience and neuroaesthetics) (Shukla et al., 2022). The field of cognitive neuroscience, including neuroaesthetics and neuroarts, has a stronger research base, more resources, and dedicated institutes for research which may offer opportunities for collaborations with art therapy researchers (King and Kaimal, 2019; King and Parada, 2021).

There was agreement across participants about the importance of increased awareness within the broader art therapy research community about issues identified in critical neuroscience literature: inequalities and imbalance perpetuated by research design and traditional science practices, the lack of awareness about antiracist publications in neuroscience, the lack of diversity in traditional neuroscience research, shortcomings of available neuroimaging technology, and the lack of transparency about limitations, scope of research findings, and positionality of art therapy researchers (Galán et al., 2021; Goings et al., 2023; La Scala et al., 2023; Rollins, 2021). Art therapists aim to conduct equitable, ethical, and sound research, reflective of the critical analysis of art therapy practice, profession, and research that has emerged over the past two decades (Hadley, 2013; Kaiser, 2017; Karcher, 2017; Sajnani et al., 2017). Efforts to increase discourse about the implications of dominant narratives have focused on representation of practitioners within the field and within professional organizations, and there has been increased awareness and transparency in understanding the cultural contexts of art therapy clients and research participants (Hughes et al., 2024). However, the field of art therapy continues to face a lack of diversity within practice, education, and research domains (Awais and Yali, 2015; Elkins and Deaver, 2015; Hughes et al., 2024). The lack of diversity within the community of art therapy neuroscience researchers is reflected in the current study which underscored recent literature calling for intentional measures to increase diverse perspectives and report on diversity in research generally (Hughes et al., 2024).

**Figure 1:**
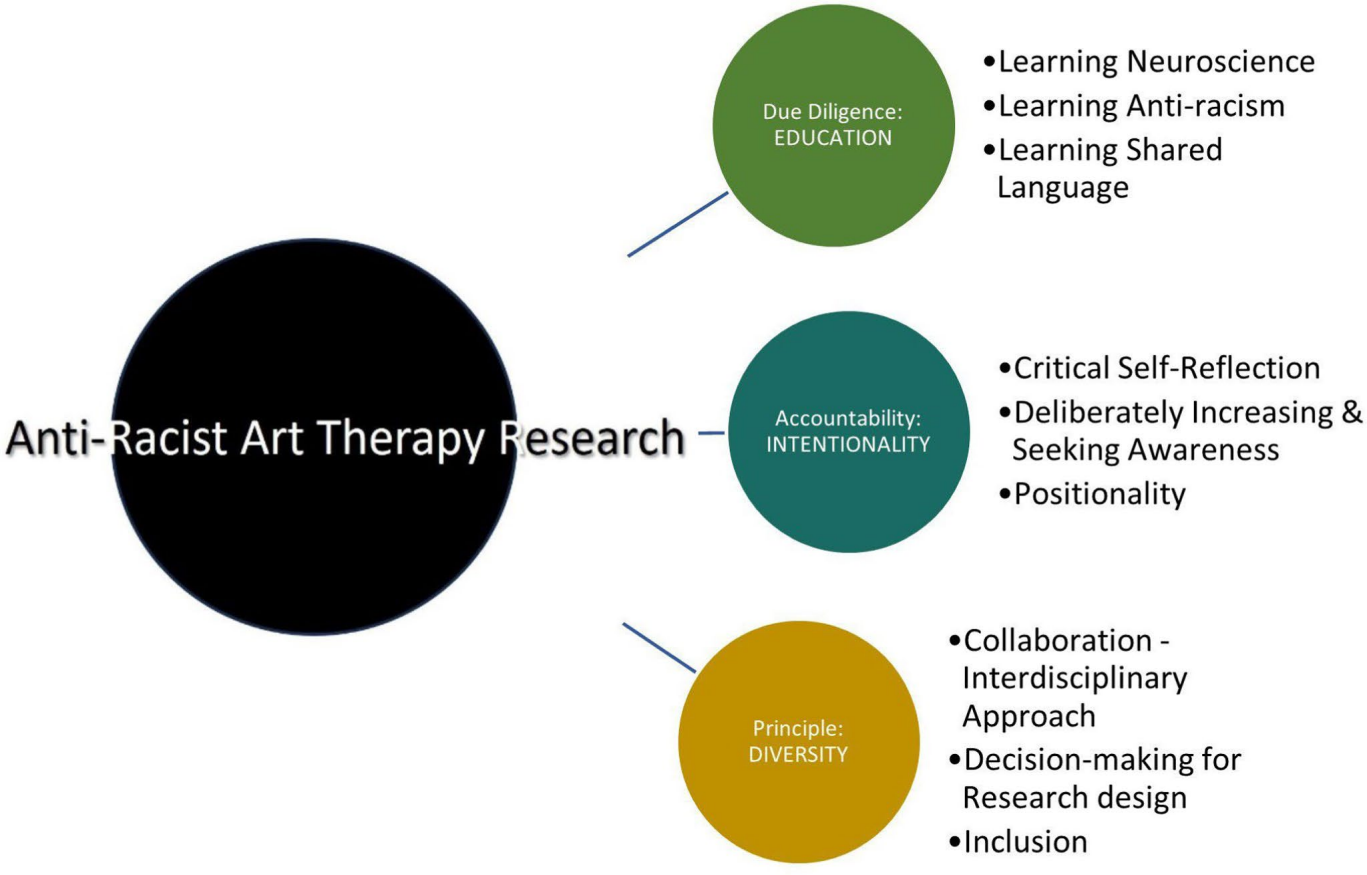
Antiracist framework of principles for art therapy research.

In response to the research question, the resulting themes under the third core category *requirements and responsibilities for antiracist research* reflected potentially important points, or perhaps principles, that specified behaviors, attitudes, and actions for antiracist neuroscience research in art therapy, but also for all research in art therapy. More research involving a greater number of art therapy researchers may be beneficial to further clarify, and outline actions needed to reflect antiracist research practices. Antiracist discourse can confront and provoke discomfort (Kendi, 2023), but by fully understanding the challenges and barriers that prevent socially-just advancement of the profession of art therapy, innovative solutions can be developed and implemented to create systemic change (Griffith and Semlow, 2020). Ultimately, the themes within this category illuminated a preliminary framework for antiracist research approaches in art therapy neuroscience studies involving due diligence through education, intentional and deliberate actions required to enhance accountability and legitimacy, and a principle of diversity is suggested to inform and underlie the research approach (see Figure 1).

### 4.2 Antiracist framework for art therapy neuroscience research

The advancement of antiracist praxis, pedagogy, and research has been espoused and examined within several adjacent and related disciplines of healthcare, education, public health, mental health, neuroscience, and other scientific disciplines (Allen et al., 2023; Chopra and Tsong, 2023; Perez, 2021; Rollins, 2021; Thomas et al., 2021; Welton et al., 2018). Across psychology and adjacent fields, there have been calls for deliberate and sweeping changes to all aspects of human services to counter racism by dismantling systems that perpetuate racial hierarchies, and to directly address oppression and oppressive practices (Awad et al., 2024). There is a need for structured approaches, theoretical frameworks, and especially treatment and research methodologies that promote equality and antiracism. Reflective of neuroscience research findings that purport racism and discrimination are underlying causes of health inequities (Berger and Sarnyai, 2015), and that implicit biases may impact research (Rollins, 2021), new frameworks to reposition the importance of critical self-awareness and intentional consideration of inclusion of diversity may facilitate equitable adaptations across research practices (Abiodun, 2019; Goings et al., 2023). The proposed framework for antiracist art therapy research (Figure 1) reflects potential principles, namely education, intentionality, and diversity, and practices that require diligence, convey accountability, and broaden perspectives necessary for ethical and equitable art therapy research.

#### 4.2.1 Due diligence: education

From their multilayered perspectives, the participants presented suggestions to enhance art therapy education curriculum designed to heighten awareness about antiracist research. To address inequalities and oppression within many decades of prior research, diligent learning through antiracist and transgressive education may facilitate structural and perceptual change (Karcher, 2017; Talwar, 2010). The review of literature about antiracism in neuroscience prompted this study. Therefore, such literature review may be beneficial within higher education and professional development to enhance awareness relevant to art therapy research. Further, a review of the APA Race and Ethnicity Guidelines (APA Task Force on Race and Ethnicity Guidelines in Psychology, 2019) is recommended for teaching psychological research which would encompass requirements for art therapy research. Fundamental education about neuroscience principles and research conducted to date might promote greater understanding of how theoretical principles require basic, empirical research to bolster the field, and also to understand shortcomings of that research due to the racist inequalities therein (Webb et al., 2022a, 2022b). For instance, antiracist art therapy neuroscience research education might include discourse about scientific racism, structural oppression, and racism throughout neuroscience research to decipher what a paradigm of antiracism might entail (Awais and Yali, 2015; Rollins, 2021). Participants also cautioned how research and subsequent publication influence both education and the practice of art therapy, and therefore recommended educators learn how to understand, integrate, and instruct within and about an antiracist paradigm by confronting their own held beliefs, lack of awareness, and comfort level to provide this instruction (Chopra and Tsong, 2023; Perez, 2021; Roth and Gavin, 2021). The first steps may include raising awareness and elevating critical consciousness through modeling, supporting cultural knowledges and identities within the classroom, acknowledging racism, encouraging activism, and critical evaluation of curricula (Chopra and Tsong, 2023; Goings et al., 2023; Roth and Gavin, 2021).

#### 4.2.2 Accountability: intentionality

Deliberate and intentional actions to enhance equitability and incorporate diversity were suggested to support antiracist art therapy neurobiological research, and may “revolutionize the very ethical bonds of accountability between (neuro)science and society” (Rollins, 2021, pg. 540). Findings from this preliminary investigation appeared to align with prior literature espousing properties of a proposed antiracist framework for research, as depicted from a social work lens, which included critical self-reflection to examine how researcher identity and various frames of reference impact decisions throughout the research process (Goings et al., 2023; La Scala et al., 2023). Scientists have lauded objectivity and impartiality as the golden standard throughout research, not as a quality of neutrality, but rather to limit intrusion of the researchers’ biases into their work (National Academies of Sciences, Engineering, and Medicine, 2017). Yet, there has been contemplation that true objectivity cannot exist without subjectivity because researchers interpret information using their own cognitive processes which shape their way of knowing (Levitt et al., 2022). Therefore, a researcher’s identity, background, experiences, perspectives, and motivations become interesting factors which position the framework, assumptions, and priorities of the research and the research question. Rice et al. (2019), purported “reflexivity disrupts power relations embedded in acts of naming and narrating others from the top down and allows space for research to be understood as a dynamic process that transforms researchers and participants” (p. 415). Specifically, participants in this current study contemplated the benefits of intentional self-reflection and critical self-awareness practices which could reveal possible biases that may influence research design and interpretation of research findings. Suggestions included invoking a critical examination of the impetus and origin of their research interests, acknowledgement and presentation of their own positionality, and heightened awareness about prior flaws and their own racist practices throughout research. Art therapy neuroscience research aims to examine the mechanisms and impact of art therapy processes and interventions which facilitate therapeutic change. To understand aspects of therapeutic change, research participants’ experiences may be necessary to examine along with considerations of their socio-cultural contexts and lived experiences. By situating the positionality of the researcher and centering contextual information of the participants recruited for research, areas of convergence and imbalance (across power and hierarchy insinuated in human subjects’ research) may be more clearly delineated (La Scala et al., 2023). Consequently, findings from this current study insinuated the importance of intentional practices to increase self-awareness, to expand diversity, and to transparently report on research participants which reflect an antiracist art therapy research approach. Therefore, critical self-reflection could become important for art therapy research education and art therapy neuroscience research design development and might include development of statements of researcher positionality to enhance accountability. These practices also align with an intersectional framework which incorporates critical awareness, critical examination of knowledge and supports empowerment to combat systems of oppression (Kuri, 2017; Talwar, 2010).

#### 4.2.3 Principle: diversity

It was inferred from this study that antiracist art therapy neuroscience research practices infuse diversity. Participants in this study reflected on concerns about the lack of diversity in the professional field and across human subjects’ research (i.e., issues of diversity amongst participant samples and recruitment), and two points arose as potentially important to antiracist research practices in art therapy research, specifically raising awareness and incorporating diversity. Diversity has been positioned as ethically necessary for critical re-examination of research practices and amongst the workforce (Thomas et al., 2021). Across the field of art therapy, there has been discourse about both the requirement to ensure social justice through inclusion (George et al., 2021), and the lack of diversity within the professional field which has perpetuated dominant narratives (e.g., White and female) and marginalization within research, education, and practice initiatives (Awais and Yali, 2015; Hadley, 2013; Talwar, 2010). Actions related to raising awareness through education and critical self-reflection may be important intentional practices for art therapist professional development, while consideration of diversity was touted as a potential principle to guide research in its entirety. By prioritizing diversity as a principle, intersectionality, positionality, and intentionality may be innately incorporated into research practices. Certainly, prioritizing diversity as a principle may be met with challenges as systemic change will also require innovations in technologies (King and Kaimal, 2019; King and Parada, 2021; Parker and Ricard, 2022; Webb et al., 2022a), expanded efforts to engage marginalized and minoritized groups (Green et al., 2022; La Scala et al., 2023; Louis et al., 2022; Wig et al., 2024), and new ways of presenting and examining contextual factors in data analysis (Goldfarb and Brown, 2022; Kwasa et al., 2023). However, diversity can be infused at all levels of the research process. Considering contributions from cognitive neuroscience, genetics, and public health, emphasizing diversity within health-related research might expand findings and knowledge. In alignment with antiracist neuroscience literature, intentional and deliberate actions are proposed to prioritize recruitment of diverse research participants through community-based engagement for partnerships and to work with knowledgeable experts in neuroimaging technology who incorporate innovative solutions to support diverse participant samples (Girolamo et al., 2022; La Scala et al., 2023; Parker and Ricard, 2022; Ricard et al., 2023; Webb et al., 2022a, 2022b). Findings in this study included reflections on the importance of collaborative and interdisciplinary approaches with like-minded researchers interested in investing energy and resources into studying the impact of artmaking on human physiological and neurobiological processes. Considered mutually beneficial, collaboration amongst a diverse, interdisciplinary research team might serve to strengthen knowledge development across disciplines and provide a variety of perspectives to enhance accountability for critical examination of the research process and decision-making in research design and method (Thomas et al., 2021).

### 4.3 Limitations

This study focused, primarily, on art therapy researchers who have integrated art therapy and neuroscience research which is a small focus within the greater field of art therapy research. The work to understand the scope of the problem of racism within neuroscience research also informed my own understanding of how to frame antiracist neuroscience research. Providing the articles prior to the scheduled interviews could be considered as potentially leading, however the step was included to highlight aspects in neuroscience literature which may not have been widely known due to the lack of publications about antiracist research within art therapy literature. The small sample of four participants, while a cross-section of international art therapists who have conducted such research, provided a potentially limited variety of perspectives and responses. Therefore, the findings from this study are preliminary limiting transferability. It would be interesting to consider responses from a larger sample size to determine if the synergy of viewpoints, noted in this study, is indicative across art therapy researchers. A larger sample size may also reveal contextual factors not considered in the present study and may enhance transferability. There is another possible limitation as my own identity aligned very closely, in several ways, with the participants. Though the data analysis included reflexive practice and intentional bracketing of my own perspectives, the solo effort in ranking the importance of components of the coding structure could reflect a narrowed scope and potentially biased thinking. Likely having a diverse team of coders at all stages of the analysis process might have created opportunities for triangulation and consensus. Further, there are limitations from my own positionality as a White, American, Protestant, heterosexual, highly educated, able-bodied, and fully employed female. While I endeavored to conduct this research to advance antiracist discourse within art therapy, and considering the growing body of antiracist literature from other fields, I fully acknowledge the limitations from my own position.

### 4.4 Recommendations

Intentional implementation of the potential framework for antiracist research practices within art therapy neuroscience research will be important to discern its applicability. Intentional, critically reflexive, and collaborative antiracist research can disrupt and dismantle oppressive stereotypes that dominate culture, medicine, communities, and divisive rhetoric (Goings et al., 2023). Recommendations that have materialized from this study suggest that art therapy students, educators, practitioners, and researchers may enhance their understanding from intentional examination of literature across disciplines that describe and outline antiracist praxis, pedagogy, and research initiatives. It is possible that educators within art therapy have not considered how to infuse antiracist practices into research curricula, primarily because there is no literature about antiracist research within art therapy. Art therapy research educators might benefit from training in antiracist education, critical reflexivity, and antiracist research practices (Awais and Yali, 2015). There have been publications describing efforts within undergraduate neuroscience and graduate medical education to infuse information about antiracism to raise critical consciousness through interdisciplinary and synchronous examinations of structural racism in neuroscience and healthcare (Godley et al., 2020; Roth and Gavin, 2021). Further research examining the impact of expanding awareness of scientific racism and antiracist frameworks on art therapy students might be instrumental in the educational development of socially-just art therapy researchers. It is invigorating to consider research at the intersection of social cognitive neuroscience and art therapy which investigates the potential for transformative and therapeutic art processes to mitigate racial biases in mental health, to address generational trauma from systemic and structural racism, and to mitigate implicit biases of researchers, students, and practitioners within art therapy.

### 4.5 Conclusion

Neuroscientific research in art therapy applies social cognitive research theories and findings to further elucidate or to demonstrate the therapeutic mechanisms of creative and expressive approaches to mental health transformation, integration, and healing (International Art Therapy Neuroscience Collective, 2025). Informed by literature describing institutionalized racism in neuroscience research and antiracist frameworks to address and disrupt systemic inequalities (Girolamo et al., 2022; Goings et al., 2023; Ricard et al., 2023), this qualitative pilot study aimed to conceptualize antiracist research practices for future art therapy research from perspectives of art therapy researchers. Within three core categories, shared beliefs and values, barriers and challenges, requirements and responsibilities for antiracist research, themes described areas of convergence amongst the participants, detailed realistic challenges facing the field of art therapy, and provided a preliminary framework for antiracist research through intentional, critically reflexive, collaborative, and diligent actions based on a principle of diversity. Participants highlighted the importance of neurobiological research for the advancement of the art therapy profession and understood the problems within traditional neuroscience design which had not emphasized diversity of participants, positionality of the researcher, and equitable technologies (Girolamo et al., 2022; Goldfarb and Brown, 2022; La Scala et al., 2023). Participants increased their awareness by engaging in review of relevant literature and discourse to emphasize incorporation of diversity in research design, recruitment, and development of collaborative and interdisciplinary teams (Hughes et al., 2024; La Scala et al., 2023). Further, participants contemplated intentional and diligent behaviors which might strengthen knowledge of neuroscience principles to enhance collaborative partnerships, self-reflective activities to enhance awareness of the impact of researcher identity on research practices and findings, and the responsibility to increase transparency through research publication that include demographics and contextual factors of participants (Goings et al., 2023; Rollins, 2021). These efforts, though challenging, reflect the proposed framework for antiracist art therapy research which focuses on feasible and actionable steps and aligns closely with the antiracist discourse within neuroscience and social work (Girolamo et al., 2022; Goings et al., 2023; La Scala et al., 2023). Potentially, a movement toward antiracist science requires expanded awareness and incorporation of a perceptual shift toward intentional inclusion, equitability, diversity, critical reflexivity, and interdisciplinary collaborations to facilitate evolution of scientific research practices.

Antiracist art therapy research practices highlight and acknowledge difference, focus on the needs of the communities who benefit from art therapy treatment, and bring together interdisciplinary partners necessary for bolstering neurobiological research that underpins and fortifies art therapy theories through equitable research practices. Antiracist experimental art therapy neuroscience research pulls from interdisciplinary perspectives and intentionally disrupts oppressive structures and may potentially mitigate implicit biases (Garcia-Arch et al., 2020). By infusing antiracist practices into neurobiological research in art therapy, findings may lead to transformative change in the field and could more aptly address health disparities for the benefit of marginalized and minoritized communities (Allen et al., 2023; Awad et al., 2024; Kubota, 2024; Kuri, 2017). By operationalizing specific antiracist research practices, art therapy neuroscience researchers may begin to employ these practices and strategies to test the framework and contribute critically informed research methodologies. Further, antiracist and equitable research strategies infused in research designed to generate scientific evidence for the foundational theories and tenets of art therapy would result in an equitable understanding of the impact of art therapy. Limitations and potential future research were also presented.

## Data Availability

The raw data supporting the conclusions of this article will be made available by the authors, without undue reservation.
