# Supplementary material for: Conceptualizing an antiracist framework for neuroscience research in art therapy: a qualitative pilot study
Source: Front Hum Neurosci. 2025 Apr 23;19:1492779. doi: 10.3389/fnhum.2025.1492779 (PMC12055810; doi:10.3389/fnhum.2025.1492779)
Supplement: Supplementary file 1 [file Data_Sheet_1.pdf]

Supplementary Item 1

Interview Guide

The interviewer will provide a brief introduction and remind the participant about Informed Consent and their right to withdraw from the study at any time.

The interviewer will provide a positionality statement:
"I am a middle-aged, White, female, English-speaking, able-bodied, American art therapist and doctoral student who has engaged in neuroscience research and I have elected to study, from a critical, postmodern perspective, how art therapy research practices have been perpetuating racism in neuroscience research and how a community of art therapy neuroscience researchers may conceptualize anti-racist neuroscience research practices."

1. Can you briefly introduce yourself and include a description of your experiences with developing research methodology for neuroscience research relevant for art therapy?
    a. [If not covered]: Can you specifically note important considerations for art therapy neuroscience research design?
2. How did you conceptualize the role of the participants and criteria for inclusion / exclusion?
3. Can you describe your responses to, or any points that particularly stuck out to you related to the provided articles?
4. [If not specifically noted]: Have you created a positionality statement that may identify areas of your perspective and potential biases as a researcher?
    a. If so, could you share it and explain its significance in your work?
    b. If not, can you share your understanding of what would be important to include in your positionality statement?
5. Can you describe the benefits of using a positionality statement in neuroscience research?
6. What would a commitment to anti-racist research entail in neuroscience research relevant to art therapy?
7. How does this impact your thoughts on research design?
8. Is anti-racist research feasible in art therapy related neuroscience research and what would be required to magnify this conversation?
9. Is there anything else you'd like to share, or anything I didn't ask you?
    a. [If additional prompting needed]: Any additional insights on systemic racism in neuroscience research or anti-racist research in art therapy neuroscience research?
